# Hyperspectral Imaging of Adaxial and Abaxial Leaf Surfaces as a Predictor of Macadamia Crop Nutrition

**DOI:** 10.3390/plants12030558

**Published:** 2023-01-26

**Authors:** Anushika L. De Silva, Stephen J. Trueman, Wiebke Kämper, Helen M. Wallace, Joel Nichols, Shahla Hosseini Bai

**Affiliations:** Centre for Planetary Health and Food Security, School of Environment and Science, Griffith University, Nathan, QLD 4111, Australia

**Keywords:** fertiliser, hyperspectral imaging, macadamia, *Macadamia integrifolia*, mineral nutrient, partial least squares regression (PLSR)

## Abstract

Tree crop yield is highly dependent on fertiliser inputs, which are often guided by the assessment of foliar nutrient levels. Traditional methods for nutrient analysis are time-consuming but hyperspectral imaging has potential for rapid nutrient assessment. Hyperspectral imaging has generally been performed using the adaxial surface of leaves although the predictive performance of spectral data has rarely been compared between adaxial and abaxial surfaces of tree leaves. We aimed to evaluate the capacity of laboratory-based hyperspectral imaging (400–1000 nm wavelengths) to predict the nutrient concentrations in macadamia leaves. We also aimed to compare the prediction accuracy from adaxial and abaxial leaf surfaces. We sampled leaves from 30 macadamia trees at 0, 6, 10 and 26 weeks after flowering and captured hyperspectral images of their adaxial and abaxial surfaces. Partial least squares regression (PLSR) models were developed to predict foliar nutrient concentrations. Coefficients of determination (R^2^_P_) and ratios of prediction to deviation (RPDs) were used to evaluate prediction accuracy. The models reliably predicted foliar nitrogen (N), phosphorus (P), potassium (K), calcium (Ca), copper (Cu), manganese (Mn), sulphur (S) and zinc (Zn) concentrations. The best-fit models generally predicted nutrient concentrations from spectral data of the adaxial surface (e.g., N: R^2^_P_ = 0.55, RPD = 1.52; P: R^2^_P_ = 0.77, RPD = 2.11; K: R^2^_P_ = 0.77, RPD = 2.12; Ca: R^2^_P_ = 0.75, RPD = 2.04). Hyperspectral imaging showed great potential for predicting nutrient status. Rapid nutrient assessment through hyperspectral imaging could aid growers to increase orchard productivity by managing fertiliser inputs in a more-timely fashion.

## 1. Introduction

Demand for food crop production is increasing rapidly with an expanding human population [1,2,3]. Tree crops currently provide over 600 million tons of the 10,600 million tons of global food production [4,5,6]. However, improving the yield and quality of tree crop products is dependent on maintaining sufficient crop nutrition [7,8,9]. Nutrient requirements within a cropping system may depend on the cultivar, climate, soil type and soil biology [10,11] and continuous monitoring of crop nutrition is often required to optimise fertiliser inputs and reduce nutrient losses [12,13,14,15]. Conventional methods for determining the crop nutrition status are generally laborious and time-consuming, creating delays between field sampling, the receipt of nutrient results, and fertiliser amendments [16,17,18]. Rapid assessment tools are needed to monitor crop nutrition in real-time, allowing growers to quickly adjust their fertiliser regimes to maximise crop productivity and reduce nutrient runoff [19,20].

Hyperspectral imaging is an emerging technology that has been adapted for the rapid assessment of soil, leaves and agricultural products [21,22,23,24,25]. Hyperspectral imaging is a combination of spectroscopic and imaging techniques that enables the identification of chemical components and their spatial distribution in a sample [26,27]. The ability to capture spatial data allows the analysis of heterogeneous samples and so hyperspectral imaging requires minimal sample preparation [27]. Spectral and spatial data acquired by the hyperspectral imaging system can be correlated with chemical concentrations in the tested sample, allowing the development of models that predict chemical composition [26].

Hyperspectral imaging is becoming an important diagnostic tool in precision agriculture for the rapid assessment of crop nutrition [19,28,29]. Hyperspectral imaging sensors mounted on platforms such as satellites, aircraft and unmanned aerial vehicles (UAV) have been used to estimate N, P and K levels in the leaves of many crops [30,31,32]. Satellite and airborne remote-sensing techniques may not always provide high-accuracy predictions of crop nutrition, as atmospheric conditions can affect prediction accuracy [33,34]. Laboratory-based hyperspectral imaging, on the other hand, has been used to predict foliar nutrient concentrations with high accuracy in apple (*Malus domestica*) (e.g., N: R^2^ = 0.77) and cacao (*Theobroma cacao*) (e.g., N: R^2^ = 0.75; P: R^2^ = 0.71) [28,35]. Prediction of foliar mineral nutrient concentrations through laboratory-based hyperspectral imaging has generally been performed using the adaxial (top) surface of leaves [19,29,36]. Models developed using spectral data from the adaxial surface of citrus (*Citrus × sinensis*) leaves provide higher accuracy (R^2^ = 0.90) in predicting N concentrations than models using the abaxial (bottom) surface (R^2^ = 0.83) [37]. The accuracy of hyperspectral imaging in predicting foliar nutrient concentrations has not been compared between the two leaf sides in other tree crops.

Macadamia (*M. integrifolia*, *M. tetraphylla* and hybrids) is the seventh largest of the tree-nut industries, accounting for 62,000 tons of the 5.3 million tons of annual tree-nut production [38]. The importance of macadamia crop nutrition for pollination success, fruit set and fruit development means that continuous monitoring of tree nutrition is critical for maintaining orchard productivity [39,40]. Timely fertilizer management leads to optimal yield and nut quality [41]. Generally, foliar nutrient levels are used as an indicator of crop nutrition status in macadamia [41,42,43]. Traditional nutrient analysis methods are time-consuming and, therefore, growers are unable to make rapid decisions to amend fertiliser applications [16,17,18]. Rapid assessment of macadamia crop nutrition could help growers to increase yield and nut quality by quickly adjusting fertilizer regimes. A multi-spectral UAV system has been used to assess visual categories of macadamia canopy conditions, without assessing tree nutrition [44]. In this study, we aimed to: (a) evaluate the capacity of laboratory-based hyperspectral imaging to predict nutrient concentrations in macadamia leaves; and (b) compare the accuracy in predicting foliar nutrient concentrations between images captured of the adaxial and abaxial leaf surfaces.

## 2. Results

### 2.1. Reflectance of Adaxial and Abaxial Surfaces

**Figure 1:**
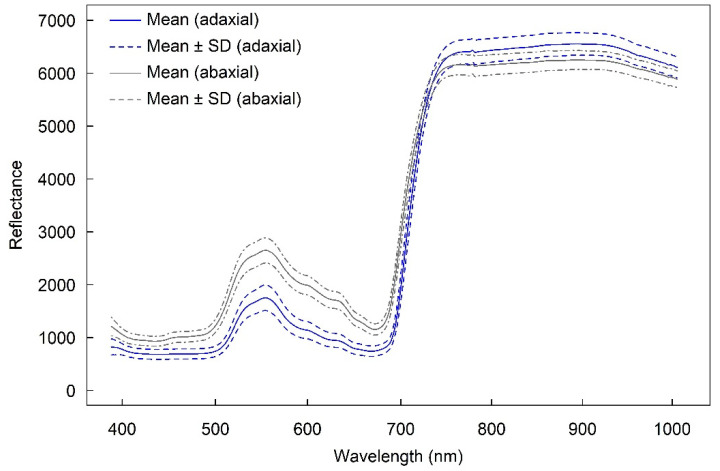
The mean (± SD) relative reflectance of the Vis–NIR spectra (400–1000 nm) from the adaxial (blue lines) and abaxial (grey lines) leaf surfaces (n = 120). The 100% reflectivity was scaled to 10,000 (integers) by default.

The adaxial and abaxial surfaces had similar spectral patterns in the 400–1000 nm wavelength region (Figure 1). However, the reflectance of the abaxial surface was higher than the adaxial surface between approximately 400 and 730 nm, and reflectance of the adaxial surface was higher than the abaxial surface between approximately 740 and 1000 nm. The highest difference in reflectance between the two surfaces was around 567 nm. The average reflectance of both the abaxial and adaxial surfaces was high at approximately 560 nm and from approximately 700 to 1000 nm. Two small peaks were observed approximately at 600 nm and 640 nm.

### 2.2. Predicting the N, P, K and Ca Concentrations

**Figure 2:**
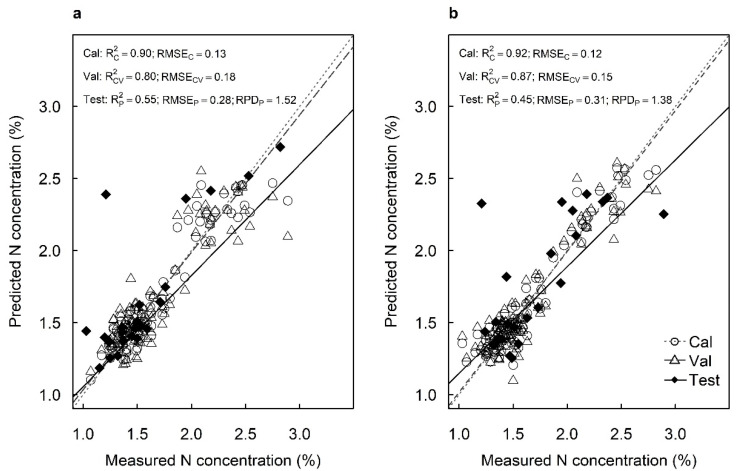
Measured vs. predicted nitrogen concentration (%) of the calibration set (Cal: open circles), cross-validation set (Val: open triangles) and test set (Test: closed diamonds) of macadamia cultivar ‘816’ leaves using hyperspectral images of the (**a**) adaxial and (**b**) abaxial surfaces and using all available wavelengths.

The best-fit model using spectral data from the adaxial surfaces predicted N concentrations with R^2^_C_ = 0.90, RMSE_C_ = 0.13%, R^2^_V_ = 0.80 and RMSE_V_ = 0.18% in the cross-validation (Figure 2a). The best-fit model using spectral data from the abaxial surfaces predicted N concentrations with R^2^_C_ = 0.92, RMSE_C_ = 0.12%, R^2^_V_ = 0.87 and RMSE_V_ = 0.15% in the cross-validation (Figure 2b). The models predicted N concentrations with good or moderate accuracy for the test sets from the adaxial surfaces (R^2^_P_ = 0.55, RMSE_P_ = 0.28% and RPD = 1.52) and the abaxial surfaces (R^2^_P_ = 0.45, RMSE_P_ = 0.31% and RPD = 1.38), respectively (Figure 2). Models developed from adaxial surfaces provided higher RPDs for predicting foliar N concentrations than those using abaxial surfaces.

**Figure 3:**
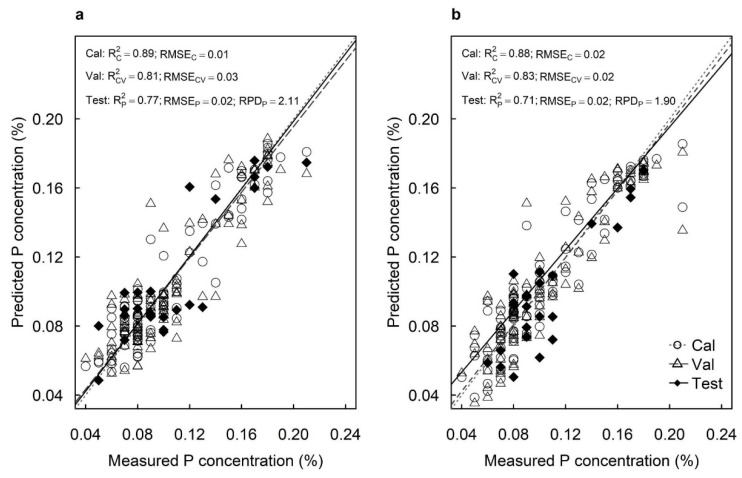
Measured vs. predicted phosphorus concentration (%) of the calibration set (Cal: open circles), cross-validation set (Val: open triangles) and test set (Test: closed diamonds) of macadamia cultivar ‘816’ leaves using hyperspectral images of (**a**) adaxial and (**b**) abaxial surfaces and using all available wavelengths.

The best-fit model using spectral data from the adaxial surfaces predicted P concentrations with R^2^_C_ = 0.89, RMSE_C_ = 0.01%, R^2^_V_ = 0.81 and RMSE_V_ = 0.03% in the cross-validation (Figure 3a). The best-fit model using spectral data from the abaxial surfaces predicted P concentrations with R^2^_C_ = 0.88, RMSE_C_ = 0.02%, R^2^_V_ = 0.83 and RMSE_V_ = 0.02% in the cross-validation (Figure 3b). The models predicted P concentrations with high accuracy for the test sets from both the adaxial surfaces (R^2^_P_ = 0.77, RMSE_P_ = 0.02% and RPD = 2.11) and abaxial surfaces (R^2^_P_ = 0.71, RMSE_P_ = 0.02% and RPD = 1.90) (Figure 3). Models developed from adaxial surfaces also provided higher RPDs for predicting foliar P concentrations than those using abaxial surfaces.

**Figure 4:**
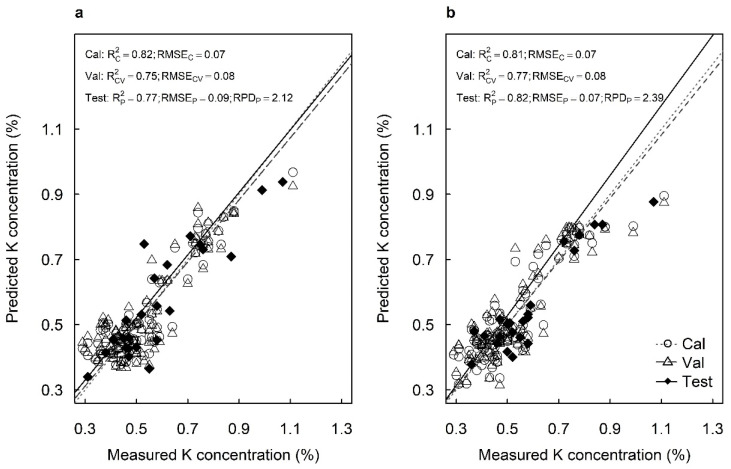
Measured vs. predicted potassium concentration (%) of the calibration set (Cal: open circles), cross-validation set (Val: open triangles) and test set (Test: closed diamonds) of macadamia cultivar ‘816’ leaves using hyperspectral images of (**a**) adaxial and (**b**) abaxial surfaces and using all available wavelengths.

The best-fit model using spectral data from the adaxial surfaces predicted K concentrations with R^2^_C_ =0.82, RMSE_C_ = 0.07%, R^2^_V_ = 0.75 and RMSE_V_ = 0.08% in the cross-validation (Figure 4a). The best-fit model using spectral data from the abaxial surfaces predicted K concentrations with R^2^_C_ = 0.81, RMSE_C_ = 0.07%, R^2^_V_ = 0.77 and RMSE_V_ = 0.08% in the cross-validation (Figure 4b). The models predicted K concentrations with high accuracy for the test sets from both the adaxial surfaces (R^2^_P_ = 0.77, RMSE_P_ = 0.09% and RPD = 2.12) and abaxial surfaces (R^2^_P_ = 0.82, RMSE_P_ = 0.07% and RPD = 2.39) (Figure 4). That is, the models developed using adaxial and abaxial surfaces provided similar RPDs in predicting foliar K concentrations.

**Figure 5:**
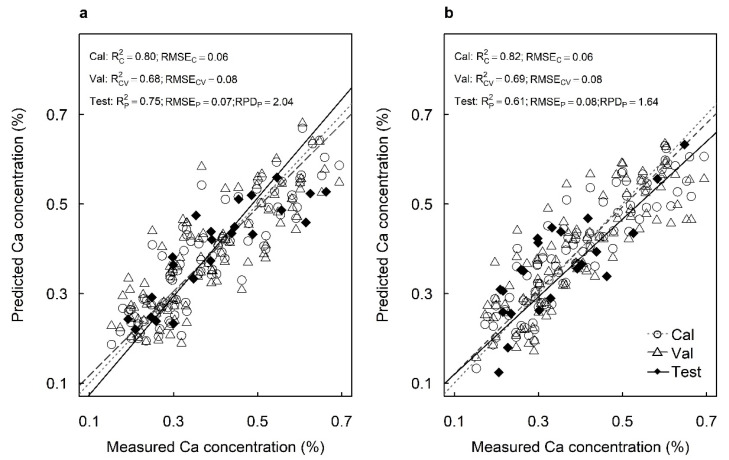
Measured vs. predicted calcium concentration (%) of the calibration set (Cal: open circles), cross-validation set (Val: open triangles) and test set (Test: closed diamonds) of macadamia cultivar ‘816’ leaves using hyperspectral images of (**a**) adaxial and (**b**) abaxial surfaces and using all available wavelengths.

The best-fit model using spectral data from the adaxial surfaces predicted Ca concentrations with R^2^_C_ = 0.80, RMSE_C_ = 0.06%, R^2^_V_ = 0.68 and RMSE_V_ = 0.08% in the cross-validation (Figure 5a). The best-fit model using spectral data from the abaxial surfaces predicted Ca concentrations with R^2^_C_ = 0.82, RMSE_C_ = 0.06%, R^2^_V_ = 0.69 and RMSE_V_ = 0.08% in the cross-validation (Figure 5b). The models predicted Ca concentrations with high accuracy for the test sets of the adaxial surface (R^2^_P_ = 0.75, RMSE_P_ = 0.07% and RPD = 2.04) (Figure 5a). However, the model predicted Ca concentrations with only moderate accuracy for the test set from the abaxial surfaces (R^2^_P_ = 0.61, RMSE_P_ = 0.08% and RPD = 1.64) (Figure 5b).

### 2.3. Predicting Other Mineral Nutrient Concentrations

The best-fit models using spectral data from the adaxial surfaces had high prediction accuracies for concentrations of Cu (R^2^_P_ = 0.80, RMSEP = 7.87 mg/kg and RPD = 2.27), Mg (R^2^_P_ = 0.95, RMSEP = 237 mg/kg and RPD = 1.31) and Zn (R^2^_P_ = 0.85, RMSEP = 1.90 mg/kg and RPD = 2.63) (Table 1). The best-fit models using spectral data from the adaxial surfaces had moderate prediction accuracies for concentrations of Mn (R^2^_P_ = 0.56, RMSEP = 40.61 mg/kg and RPD = 1.54) and S (R^2^_P_ = 0.49, RMSEP = 319 mg/kg and RPD = 1.43) (Table 1). Adaxial surfaces provided low prediction accuracies for Al, B, Fe and Na concentrations (Table 1).

The best-fit model using spectral data from the abaxial surfaces had high prediction accuracy for Cu concentration (R^2^_P_ = 0.76, RMSE_P_ = 8.19 mg/kg and RPD = 2.10) (Table 1). The best-fit models using spectral data from the abaxial surfaces had moderate prediction accuracies for concentrations of S (R^2^_P_ = 0.52, RMSE_P_ = 281 mg/kg and RPD = 1.47) and Zn (R^2^_P_ = 0.51, RMSE_P_ = 2.74 mg/kg and RPD = 1.46) (Table 1). Abaxial surfaces provided low prediction accuracies for Al, B, Mg, Mn and Na concentrations (Table 1). No model could be developed to predict Fe concentrations using spectral data from the abaxial surfaces (Table 1).

## 3. Discussion

Our study highlights the potential of hyperspectral imaging as a real-time diagnostic tool to assess macadamia crop nutrition. Hyperspectral imaging in the Vis–NIR region (400–1000 nm) had the capacity to predict N, P, K, Ca, Cu, Mn, S and Zn concentrations in macadamia leaves with RPD greater than 1.4. Prediction accuracy for each of these nutrients was generally higher using spectral data from the adaxial surface than from the abaxial surface of leaves. Rapid estimation of crop nutrition could aid macadamia growers to increase orchard productivity, minimise fertiliser costs and reduce nutrient losses by allowing more-timely and responsive fertiliser management.

Laboratory-based hyperspectral imaging predicted N, P, K, Ca, Cu, Mn, S and Zn concentrations reliably in macadamia leaves with RPD > 1.4. Models that provide RPDs between 1.4 and 2.0 are considered “good” while models with RPDs > 2.0 are considered “excellent” [45]. Mineral nutrients do not absorb light in the Vis–NIR region [27] but hyperspectral imaging can detect mineral nutrients indirectly as organic macromolecules that form bonds with mineral nutrients to maintain cellular structure and function [36]. Prediction of N, P, K, Ca, Cu, Mn, S and Zn concentrations in macadamia leaves might have been possible due to light-absorbing organic macromolecules that formed complexes with these nutrients.

Some mineral nutrient concentrations including N, B, Mn and Na were predicted with moderate or low accuracy, although the N prediction had a model robustness of RPD > 1.4. Low prediction accuracy for foliar B, Mn and Na concentrations has been reported previously from maize (*Zea mays*) and soybean (*Glycine max*) [46]. However, hyperspectral imaging has predicted foliar N concentrations with high accuracy in oilseed rape (*Brassica napus*), maize and soybean [19,46]. Nitrogen is a component of the photosynthetic enzyme, ribulose-1,5-bisphosphate carboxylase/oxygenase (rubisco), and so hyperspectral imaging might detect N indirectly as chlorophyll, which exhibits strong absorption in parts of the visible spectrum [46,47]. The data range can influence the prediction ability of hyperspectral imaging models [28,46]. The range of foliar N concentrations in the current study was 1.03–2.89% whereas the range in oilseed rape was 3.48–6.21% and in maize and soybean was 0.96–5.68% [19,46]. The narrower range of foliar N concentrations in macadamia leaves may have decreased the prediction accuracy.

Adaxial surface images were more reliable than abaxial surface images in predicting the levels of many mineral nutrients in macadamia leaves. The prediction ability for the estimation of P and Ca concentrations decreased from “excellent” to “good”, and the prediction ability for the estimation of N concentration decreased from “good” to “moderate” when we developed models using abaxial- rather than adaxial-surface data. Spectral data from the abaxial surface has approximately 10% lower accuracy than data from the adaxial surface in predicting foliar N and P concentrations of citrus leaves [37]. These differences in prediction accuracy were associated with structural differences between the adaxial and abaxial surfaces [37]. We found that spectral data from the abaxial surface of macadamia leaves had 6–14% lower accuracy than data from the adaxial surface in predicting foliar N, P and Ca concentrations. The upper epidermis and palisade parenchyma of macadamia leaves have a more-uniform cell arrangement than the lower epidermis and spongy parenchyma [48,49]. Furthermore, palisade parenchyma is chloroplast-dense and absorbs more light than spongy parenchyma [50,51]. Higher prediction performance using the adaxial surface might be explained partly by the uniform cell structure of the upper epidermis and palisade parenchyma and the higher chloroplast density of the palisade parenchyma, which could increase the stability of reflectance. Abaxial surfaces also provided “low” prediction accuracies for Al, B, Mg, Mn and Na concentrations. We, therefore, recommend scanning the adaxial surface when using hyperspectral imaging to predict nutrient levels in macadamia leaves.

## 4. Materials and Methods

### 4.1. Sample Collection and Processing

**Figure 6:**
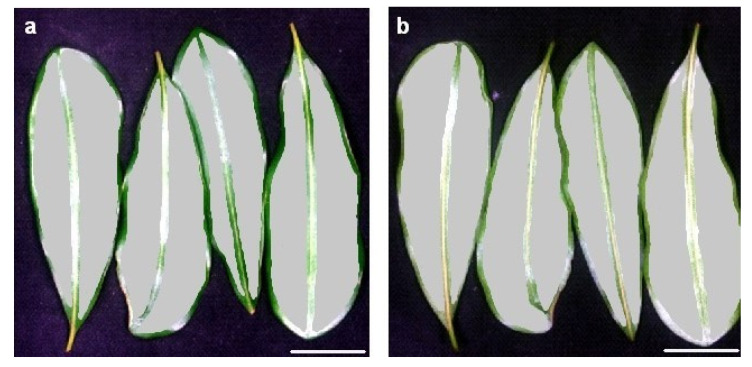
Regions of interest (shown in grey) that were used to extract mean spectral reflectance for (**a**) adaxial and (**b**) abaxial surfaces of macadamia cultivar ‘816’ leaves (scale bar = 22 mm).

The sampling site was in a commercial macadamia orchard at Alloway (24°56′6″ S 152°21′16″ E), Queensland, Australia. We selected thirty trees in a block of cultivar ‘816’ trees that were 13 years old. Tree spacing was 2 m within each row and 10 m between rows. The experimental trees had a mean height of 8.0 ± 0.1 m and trunk circumference of 63 ± 1 cm (± SE) at 10 cm above the graft union (n = 30). Each experimental tree was randomly allocated to one of three boron-fertiliser treatments (0, 15 or 30 g B per tree) that were applied prior to flowering [52]. We collected leaves from each tree on four occasions between September 2018 and March 2019, specifically at 0 weeks after peak anthesis (peak flowering), 6 and 10 weeks after peak anthesis (premature fruit drop) and 26 weeks after peak anthesis (commencement of harvesting) [53]. We collected one young fully expanded leaf from each of the four cardinal directions on each of the thirty trees at each sampling time; i.e., a total of 120 leaves at each sampling time. The leaves were transferred to the laboratory immediately after collection. The four leaves from each tree at each sampling time were pooled to constitute a single sample for imaging and mineral nutrient analysis. We captured two images from each sample of four leaves: (1) the adaxial surfaces; and (2) the abaxial surfaces (Figure 6a,b). We collected a total of 240 images, consisting of 120 images from adaxial surfaces and 120 images from abaxial surfaces; i.e., four sampling times × 30 trees × two leaf surfaces.

### 4.2. Hyperspectral Imaging System

We used a laboratory-based visible–near-infrared (Vis–NIR) hyperspectral imaging system (Pika XC2, Resonon, Bozeman, MT) for image acquisition. The imaging system had a 12-bit line-scanner camera with a lens of 23 mm focal length, four current-controlled wide-spectrum quartz-halogen lights, a linear translation stage operated by a stepper motor, and data acquisition software (Spectronon Pro Version 2.94, Resonon, Bozeman, MT, USA). The spectral resolution of the camera was ~1.3 nm. The camera captured 462 wavelengths between 400 and 1000 nm [54]. The samples were placed on a black tray on the translation stage. The scanning speed and exposure time were 1.23 mm s^−1^ and 17.57 ms, respectively.

### 4.3. Image Calibration and Spectral Profile Extraction

We used Spectronon Pro software (Version 3.2.0; Resonon, Bozeman, MT, USA) to extract the spectral data of the acquired images. The mean raw reflectance (R_0_) of each sample was extracted by marking a region of interest (ROI) for each leaf image. The ROI was selected such that it covered all four leaves in each sample (Figure 6a,b). The mean corrected relative reflectance (R) was calculated using Equation (1):

(1) $$R=\frac{\left(R_{0}-D\right)}{\left(W-D\right)}$$

where R_0_ is the mean raw reflectance, D is the reflectance of a dark image when the camera lens was covered with the lens cap (dark calibration) and W is the reflectance of a white Teflon sheet that reflected ~99% of incident light (white calibration) [55,56].

### 4.4. Mineral Nutrient Analysis

We dried the leaf samples at 60 °C immediately after imaging. The four dried leaves collected from each tree at each sampling time were ground together into a fine powder. We used approximately 300 mg subsample of the ground leaf powder to analyse their mineral nutrient concentrations. Total N concentration was determined by combustion analysis using a LECO 928 Macro Determinator (LECO, Saint Joseph, MI) [57,58]. Aluminium (Al), boron (B), calcium (Ca), copper (Cu), iron (Fe), K, magnesium (Mg), manganese (Mn), P, sodium (Na), sulphur (S) and zinc (Zn) concentrations were determined by inductively coupled plasma–atomic emission spectroscopy after nitric and perchloric acid digestion [59,60].

### 4.5. Model Development

We obtained the spectral average of the four leaves from each image and created two data sets, consisting of spectral data extracted from the images of (1) the adaxial surfaces and (2) the abaxial surfaces. Spectral outliers in each data set were detected using Hotelling’s T^2^ test with a 95% confidence interval and removed from the analysis [61]. The remaining samples were divided randomly into two groups, with approximately 80% of the samples assigned to the calibration set and 20% of the samples assigned to the test set [24]. A *t*-test was performed to confirm that the means of the calibration and test sets for each mineral nutrient in each data set were not significantly different (*p* > 0.05) (Table 2). Spectral transformations were performed on the calibration set to increase the signal-to-noise ratio and decrease the influence of undesired light-scattering effects (Figures S1 and S2) [54,62]. The applied transformations included multiplicative scatter correction (MSC), area normalisation, de-trending, orthogonal signal correction (OSC), and standard normal variate (SNV) [63,64,65,66]. Partial least squares regression (PLSR) models were developed for each mineral nutrient, using both raw and transformed data, to correlate foliar mineral nutrient concentrations with relative reflectance in the full spectral range of 400–1000 nm [67]. Partial least squares regression is particularly suitable for a data set when the number of variables is greater than the number of samples and when the predictor variables are highly correlated [68,69]. The optimal number of latent variables (LV) for establishing the calibration model was determined at the minimum value of predicted residual error sum of squares (PRESS) of the cross-validation set, using Equation (2) [70]:

(2) $$\mathrm{PRESS}=\sum_{i=1}^{n}{\left({\hat{y}}_{i}-y_{i}\right)}^{2}$$

where n is the number of samples, and ${\hat{y}}_{i}$ and $y_{i}$ are the predicted and reference values in the ith sample, respectively.

**Figure 7:**
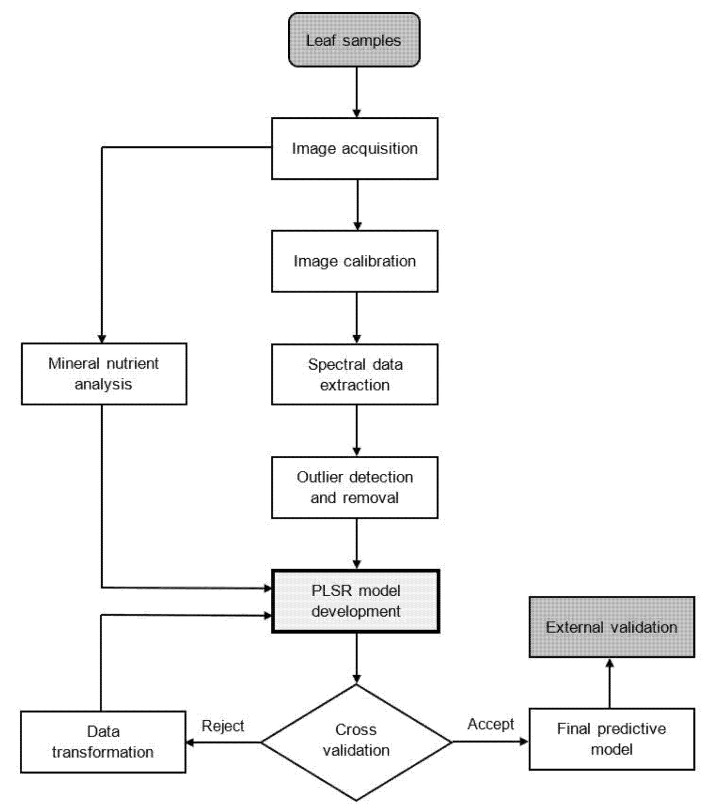
Schematic diagram of hyperspectral image analysis and the development procedure for predictive models.

We used a full cross-validation (leave-one-out) method to avoid over-fitting data and to obtain the optimum performance from the model [19,71]. The model with the highest coefficients of determination for calibration (R^2^_C_) and cross-validation (R^2^_V_) and the lowest root mean square errors for calibration (RMSE_C_) and cross-validation (RMSE_V_) was selected as the best-fit model for each mineral nutrient. The R^2^ and RMSE values were calculated using Equations (3) and (4), respectively [72]:

(3) $$R^{2}=1-\frac{\sum_{i=1}^{n}{\left(y_{i}-{\hat{y}}_{i}\right)}^{2}}{\sum_{i=1}^{n}{\left(y_{i}-\overset{-}{y}\right)}^{2}}$$

(4) $$\mathrm{RMSE}=\sqrt{\sum_{\;i=1}^{\;n}{\left({\hat{y}}_{i}-y_{i}\right)}^{2}/n}$$

where n is the number of samples, $y_{i}$ and ${\hat{y}}_{i}$ are the reference and predicted values in the ith sample, respectively, and $\overset{-}{y}$ is the mean of each reference value. The complete procedure for hyperspectral image analysis and the development of predictive models is summarised (Figure 7).

### 4.6. Evaluating Model Performance Using the External Test Set

We then assessed the prediction ability of the final model for each mineral nutrient using the test set. The ratio of prediction to deviation (RPD) was calculated for each model to evaluate its prediction ability, using Equation (5) [61,73]:

(5) $$\mathrm{RPD}=\frac{{\mathrm{SD}}_{T}}{{\mathrm{RMSE}}_{T}}$$

where SD_T_ is the standard deviation of the reference values in the test set and RMSE_T_ is the root mean square error of the prediction from the test set. Outlier detection and removal, spectral transformations, and model development were performed using Unscrambler software (Version 10.5.1; CAMO, Oslo, Norway).

## 5. Conclusions

This study demonstrated that laboratory-based hyperspectral imaging is a promising tool for rapidly predicting mineral nutrient concentrations, particularly N, P, K, Ca, Cu, Mn, S and Zn, in macadamia leaves. Ratios of prediction to deviation (RPDs) were greater than 1.4 and coefficients of determination for prediction (R^2^_P_) ranged from 0.52 to 0.85. Spectral data from adaxial leaf surfaces was more suitable than from abaxial leaf surfaces for developing models with high accuracy and predictive performance. Our results highlight the potential of hyperspectral imaging for monitoring crop nutrient levels, which could assist growers to maximise orchard productivity through timely fertiliser management. The rapid assessment of crop nutrition may also help to minimise fertiliser costs and reduce nutrient runoff to the downstream environment.

## Figures and Tables

**Table 1:** Performance of partial least squares regression (PLSR) models in predicting mineral nutrient concentrations from the adaxial and abaxial surfaces of the leaves of macadamia cultivar ‘816’.

Nutrient	Surface	Transformation	LV	Calibration Set		Validation Set		Test Set	
				RMSE_C_	R^2^_C_	RMSE_CV_	R^2^_CV_	RPD	R^2^_P_
N (%)	Adaxial	Raw	13	0.13	0.90	0.18	0.80	1.52	0.55
	Abaxial	SNV	10	0.12	0.92	0.15	0.87	1.38	0.45
P (%)	Adaxial	Normalise	11	0.01	0.89	0.03	0.81	2.11	0.77
	Abaxial	MSC	6	0.02	0.88	0.02	0.83	1.90	0.71
K (%)	Adaxial	Raw	8	0.07	0.82	0.08	0.75	2.12	0.77
	Abaxial	SNV	5	0.07	0.81	0.08	0.77	2.39	0.82
Ca (%)	Adaxial	DTR–2	9	0.06	0.80	0.08	0.68	2.04	0.75
	Abaxial	DTR–2	11	0.06	0.82	0.08	0.69	1.64	0.61
Al (mg/kg)	Adaxial	MSC	10	26.4	0.52	32.7	0.28	1.25	0.33
	Abaxial	DTR–4	4	30.0	0.42	32.1	0.20	1.14	0.20
B (mg/kg)	Adaxial	SNV	3	49.1	0.45	52.7	0.38	1.15	0.22
	Abaxial	DTR–3	3	49.2	0.45	51.5	0.41	1.21	0.29
Cu (mg/kg)	Adaxial	DTR–2	8	6.65	0.81	7.82	0.75	2.27	0.80
	Abaxial	DTR–2	11	5.57	0.89	6.76	0.82	2.10	0.76
Fe (mg/kg)	Adaxial	MSC	1	18.6	0.03	19.0	0.01	1.03	0.01
	Abaxial	MSC	9	14.9	0.44	18.1	0.19	0.64	NA
Mg (mg/kg)	Adaxial	DTR–3	5	163	0.56	178	0.48	1.31	0.95
	Abaxial	DTR–3	11	131	0.75	177	0.55	1.18	0.25
Mn (mg/kg)	Adaxial	Normalise	5	34.2	0.49	37.7	0.39	1.54	0.56
	Abaxial	DTR–2	5	33.4	0.56	36.8	0.48	1.11	0.15
Na (mg/kg)	Adaxial	OSC	2	42.0	0.25	45.4	0.14	1.21	0.29
	Abaxial	Normalise	8	46.1	0.35	54.5	0.11	1.05	0.05
S (mg/kg)	Adaxial	DTR–3	5	314	0.45	356	0.31	1.43	0.49
	Abaxial	DTR–3	6	324	0.44	351	0.36	1.47	0.52
Zn (mg/kg)	Adaxial	MSC	13	2.36	0.80	3.16	0.64	2.63	0.85
	Abaxial	DTR–2	9	2.59	0.77	3.30	0.64	1.46	0.51

LV: optimal latent variables in the model; RMSE_C_: root mean square error of calibration; R^2^_C_: correlation coefficient of calibration; RMSE_CV_: root mean square error of cross-validation; R^2^_CV_: correlation coefficient of cross-validation; RPD: ratio of prediction to deviation; R^2^_P_: correlation coefficient of prediction; SNV: standard normal variate; MSC: multiplicative scatter correction; DTR–2: de-trending polynomial order 2; DTR–3: de-trending polynomial order 3; DTR–4: de-trending polynomial order 4; OSC: orthogonal signal correction; NA: correlation coefficient of prediction is not available.

**Table 2:** Descriptive analysis of the calibration and test sets for mineral nutrient concentrations in macadamia cultivar ‘816’ leaves.

Nutrient	Surface	Sample Set	n	Min	Max	CV	Mean	SD
N (%)	Adaxial	Calibration	96	1.07	2.89	0.24	1.68	0.41
		Test	24	1.03	2.82	0.27	1.56	0.43
	Abaxial	Calibration	96	1.03	2.82	0.25	1.64	0.41
		Test	24	1.21	2.89	0.25	1.71	0.43
P (%)	Adaxial	Calibration	96	0.04	0.21	0.39	0.10	0.04
		Test	24	0.05	0.21	0.40	0.11	0.04
	Abaxial	Calibration	96	0.04	0.21	0.40	0.11	0.04
		Test	24	0.06	0.18	0.32	0.11	0.03
K (%)	Adaxial	Calibration	96	0.29	1.11	0.30	0.53	0.16
		Test	24	0.31	1.07	0.32	0.59	0.19
	Abaxial	Calibration	96	0.29	1.11	0.31	0.53	0.16
		Test	24	0.36	1.07	0.30	0.59	0.17
Ca (%)	Adaxial	Calibration	96	0.15	0.69	0.36	0.38	0.14
		Test	23	0.19	0.66	0.34	0.40	0.14
	Abaxial	Calibration	95	0.15	0.69	0.35	0.39	0.14
		Test	22	0.21	0.65	0.36	0.35	0.13
Al (mg/kg)	Adaxial	Calibration	96	26.9	196.6	0.57	69.2	39.7
		Test	24	26.4	189.7	0.62	59.3	36.6
	Abaxial	Calibration	96	26.9	196.6	0.57	69.2	39.7
		Test	24	26.4	189.7	0.62	59.3	36.6
B (mg/kg)	Adaxial	Calibration	92	13.0	300.2	0.77	86.2	66.4
		Test	23	16.0	279.8	0.81	79.7	64.7
	Abaxial	Calibration	96	13.0	357.8	0.81	82.2	66.5
		Test	24	16.0	300.2	0.77	106.6	82.0
Cu (mg/kg)	Adaxial	Calibration	96	3.08	62.44	0.87	17.89	15.49
		Test	24	3.37	67.14	0.82	21.91	17.88
	Abaxial	Calibration	96	3.08	67.14	0.82	19.52	15.92
		Test	24	3.31	62.44	1.05	15.38	16.22
Fe (mg/kg)	Adaxial	Calibration	96	40.1	125.1	0.28	67.1	19.0
		Test	24	41.5	120.7	0.30	67.9	20.4
	Abaxial	Calibration	96	40.1	125.1	0.30	67.6	20.0
		Test	24	41.5	98.2	0.24	66.1	15.8
Mg (mg/kg)	Adaxial	Calibration	96	736	2056	0.21	1162	246
		Test	24	746	1836	0.25	1231	311
	Abaxial	Calibration	96	736	2056	0.22	1165	261
		Test	24	840	1836	0.21	1219	260
Mn (mg/kg)	Adaxial	Calibration	96	21.4	251.3	0.56	86.3	48.1
		Test	24	19.2	286.4	0.70	88.8	62.5
	Abaxial	Calibration	96	21.4	286.4	0.57	88.8	50.9
		Test	24	19.2	209.6	0.66	78.9	52.0
Na (mg/kg)	Adaxial	Calibration	94	53.1	402.7	0.41	120.3	48.8
		Test	24	59.6	349.8	0.56	148.7	82.5
	Abaxial	Calibration	96	53.1	402.7	0.46	125.2	57.7
		Test	24	75.0	349.8	0.47	129.2	60.5
S (mg/kg)	Adaxial	Calibration	96	1015	3607	0.22	1908	426
		Test	24	1152	3101	0.23	1963	456
	Abaxial	Calibration	96	1015	3607	0.23	1901	435
		Test	24	1555	3101	0.21	1992	413
Zn (mg/kg)	Adaxial	Calibration	96	7.31	30.91	0.35	15.09	5.27
		Test	23	6.51	26.31	0.37	13.67	4.99
	Abaxial	Calibration	96	6.51	30.91	0.36	15.06	5.46
		Test	22	8.74	21.21	0.29	13.72	3.99

Min: minimum; Max: maximum; CV: coefficient of variation; SD: standard deviation. Means of the calibration and test sets for each mineral nutrient do not differ significantly (student’s *t*-test, *p* > 0.05).

## Data Availability

The data presented in this study are available on request from the corresponding author.

## Supplementary Materials

The following supporting information can be downloaded at: https://www.mdpi.com/article/10.3390/plants12030558/s1, Figure S1: The relative reflectance spectra of raw and transformed data from the adaxial leaf surface; Figure S2: The relative reflectance spectra of raw and transformed data from the abaxial leaf surface.
