# Supplementary material for: Hyperspectral Imaging of Adaxial and Abaxial Leaf Surfaces as a Predictor of Macadamia Crop Nutrition
Source: Plants (Basel). 2023 Jan 26;12(3):558. doi: 10.3390/plants12030558 (PMC9921287; doi:10.3390/plants12030558)
Supplement: Supplementary file 1 [file plants-12-00558-s001.zip › plants-2088189-supplementary.pdf]

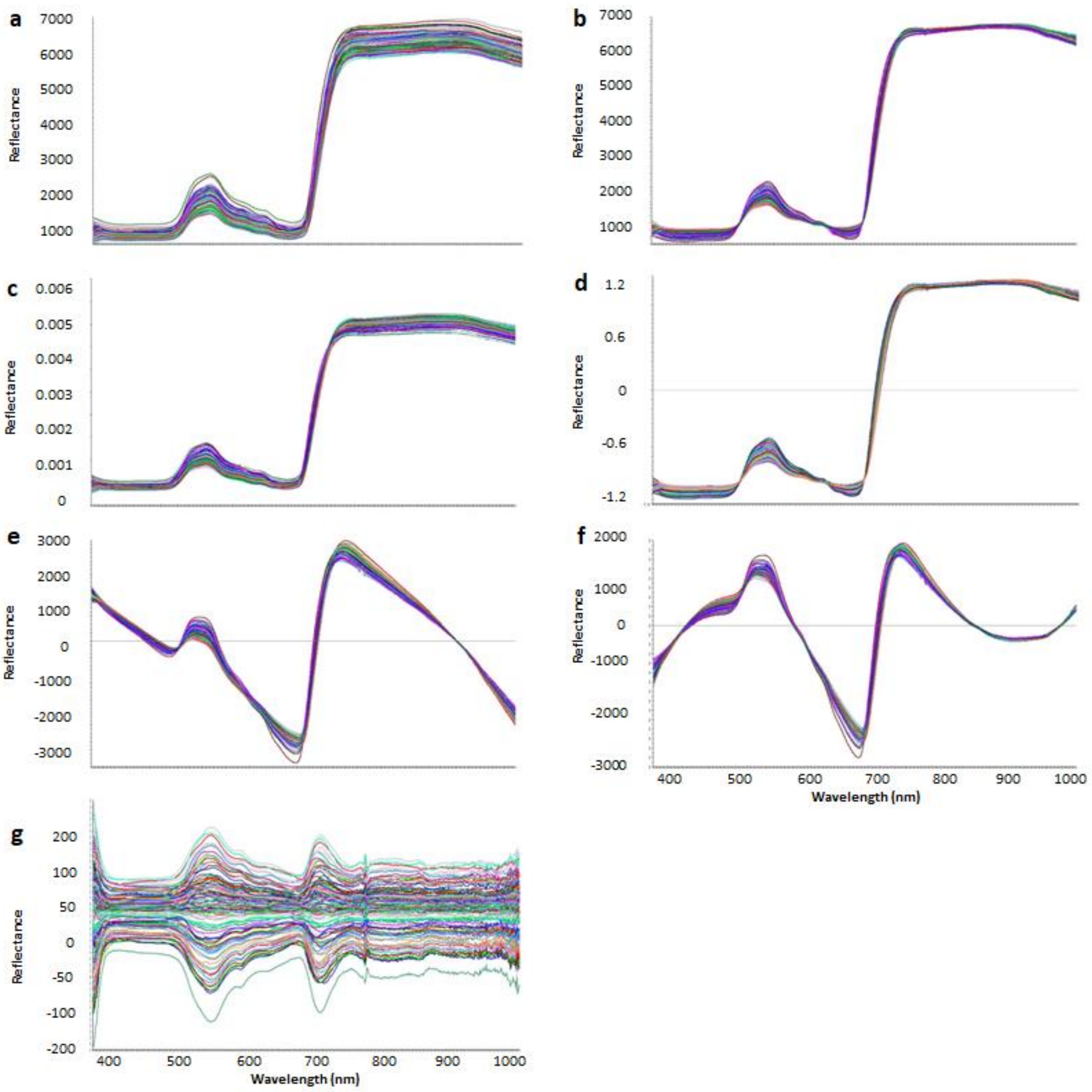

Figure S1. The relative reflectance of (**a**) raw, (**b**) MSC–transformed, (**c**) area normalised, (**d**) SNV–transformed, (**e**) detrended (polynomial order 2), (**f**) detrended (polynomial order 3) and (**g**) OSC–transformed data from the adaxial leaf surface. MSC: Multiplicative Scatter Correction; SNV: Standard Normal Variate; OSC: Orthogonal Signal Correction.

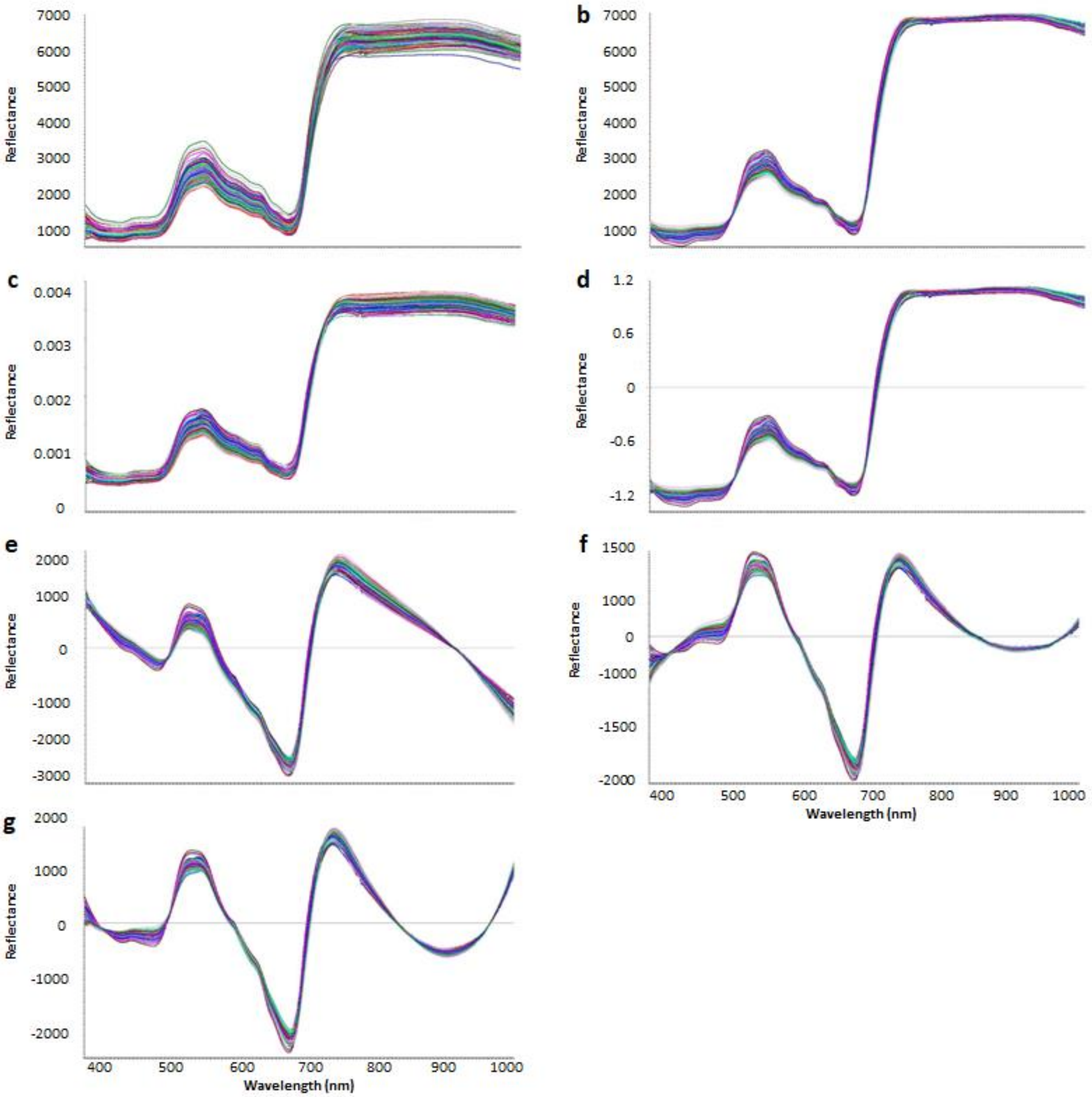

Figure S2. The relative reflectance of (**a**) raw, (**b**) MSC–transformed, (**c**) area normalised, (**d**) SNV–transformed, (**e**) detrended (polynomial order 2), (**f**) detrended (polynomial order 3) and (**g**) detrended (polynomial order 4) data from the abaxial leaf surface. MSC: Multiplicative Scatter Correction; SNV: Standard Normal Variate.
